# TRAFIC: statistical design and analysis plan for a pragmatic early phase 1/2 Bayesian adaptive dose escalation trial in rheumatoid arthritis

**DOI:** 10.1186/s13063-021-05384-5

**Published:** 2021-07-06

**Authors:** M. Cole, C. Yap, C. Buckley, W. F. Ng, I. McInnes, A. Filer, S. Siebert, A. Pratt, J. D. Isaacs, D. D. Stocken

**Affiliations:** 1 Population Health Sciences Institute, Newcastle University, Newcastle upon Tyne, UK; 2 Clinical Trials and Statistics Unit, The Institute of Cancer Research, London, Sutton, UK; 3 School of Immunity and Infection, University of Birmingham, Birmingham, UK; 4 Translational and Clinical Research Institute, Newcastle University, Newcastle upon Tyne, UK; 5 College of Medical, Veterinary and Life Sciences, University of Glasgow, Glasgow, UK; 6 Leeds Institute of Clinical Trials Research, University of Leeds, Leeds, UK

**Keywords:** CRM, Dose-finding, Adaptive, Early-phase, Clinical trial

## Abstract

**Background:**

Adaptive model-based dose-finding designs have demonstrated advantages over traditional rule-based designs but have increased statistical complexity but uptake has been slow especially outside of cancer trials. TRAFIC is a multi-centre, early phase trial in rheumatoid arthritis incorporating a model-based design.

**Methods:**

A Bayesian adaptive dose-finding phase I trial rolling into a single-arm, single-stage phase II trial. Model parameters for phase I were chosen via Monte Carlo simulation evaluating objective performance measures under clinically relevant scenarios and incorporated stopping rules for early termination. Potential designs were further calibrated utilising dose transition pathways.

**Discussion:**

TRAFIC is an MRC-funded trial of a re-purposed treatment demonstrating that it is possible to design, fund and implement a model-based phase I trial in a non-cancer population within conventional research funding tracks and regulatory constraints. The phase I design allows borrowing of information from previous trials, all accumulated data to be utilised in decision-making, verification of operating characteristics through simulation, improved understanding for management and oversight teams through dose transition pathways. The rolling phase II design brings efficiencies in trial conduct including site and monitoring activities and cost.

TRAFIC is the first funded model-based dose-finding trial in inflammatory disease demonstrating that small phase I/II trials can have an underlying statistical basis for decision-making and interpretation.

**Trial registration:**

Trials Registration: ISRCTN, ISRCTN36667085. Registered on September 26, 2014.

## Background

The development of novel therapies has brought efficient, contemporary statistical model-based dose-finding designs to assess levels of toxicity and activity. Adaptive model-based designs for dose-finding studies are based on making dose recommendations given all accumulated data at that time. They have demonstrated advantages to traditional rule-based designs, such as the 3 + 3 design [1, 2] but have increased statistical complexity with a historical lack of simulation software resulting in slow uptake. Adoption has improved within the cancer clinical trial setting but remains poor—only 1.6% of trials published 1991 to 2006 used model-based approaches [3] increasing to only 6.4% of trials published 2012 to 2014 [4]. There is a paucity of early phase adaptive designs across all fields of medicine. A systematic review in rheumatology [5] identified just one adaptive early phase design from 62 trials considered. Rheumatoid arthritis (RA) afflicts 0.5–1.0% of adults globally, presenting with coincident morbidities including vascular bone and cognitive deficits which substantially impact quality of life, disability and long-term survival. Around a third of patients have stopped working within 2 years of onset and around a half by 10 years with significant costs to the economy [6]. Advances in RA management and biologic therapies have contributed to an improved prognosis but up to 50% fail to achieve remission. Prior therapeutics have focussed primarily on immune-based therapeutics. We sought to evaluate a stromal targeting strategy, and since this is an entirely novel approach, we required alternative methods to minimise patient exposure pending estimation of safety and initial indication of potential efficacy.

In this paper, we report the statistical design, calibration and implementation of the Targeting the Rheumatoid Arthritis Synovial Fibroblast with Cyclin-Dependent Kinase Inhibition (TRAFIC) trial. TRAFIC is a non-commercial, multi-centre, phase I/II trial incorporating a Bayesian adaptive model-based dose-finding phase I design to determine the safety, tolerability and efficacy of seliciclib as an addition to existing therapy in patients with RA. Seliciclib (R-roscovitine) is an orally available cyclin-dependent kinase inhibitor with an acceptable toxicity profile [7] repurposed from the oncology setting. Determining the toxicity profile of seliciclib used in combination with a biologic plus or minus conventional synthetic disease-modifying anti-rheumatic drugs (sDMARDs) is an essential component of TRAFIC, providing important insight into its potential acceptability as an adjunctive therapy in RA. Rolling the trial from phase I to phase II allows site activity and momentum to be retained bringing trial conduct efficiencies including internal and external trial monitoring activities and associated cost savings. It also allows continuity for clinical, trial and monitoring teams. These advantages, and also potential advantages of ‘rolling’ patients from one phase to also contribute to the next, make this approach attractive when conducting challenging early phase trials.

## Methods

### Statistical design

TRAFIC has a phase I dose-finding trial rolling into a single-arm, single-stage phase II trial. The full trial protocol has been previously published [8]. The primary objective of phase I is to determine the maximum tolerated dose (MTD) of seliciclib over a 4-week treatment period when given in addition to an existing TNF inhibitor with or without sDMARDs. Phase I is planned to roll into phase II for which the primary objective is to assess the potential efficacy of seliciclib following 12 weeks of treatment when administered at the MTD established in phase I; efficacy is based on a composite response measure.

### Phase I continual reassessment method dose-finding design

The MTD, based on the occurrence of dose-limiting toxicities (DLTs), will be established using a modified one-stage Bayesian continual reassessment method (CRM) model-based design [9]. A DLT is defined as the cessation of the Investigational Medicinal Product (IMP) due to adverse events or reactions (AE/AR) occurring during the 4-week treatment period. These can be either symptomatic (e.g. nausea) or abnormal laboratory parameters or investigations. In addition, a DLT may be based upon the patient’s request to stop treatment. In the event of several AEs/ARs contributing to the decision to discontinue IMP, only a single DLT will be recorded for that participant. A flare of RA will not be considered an AE. The MTD is the dose level with an estimated DLT rate closest to the target DLT rate of 35%, i.e. the dose level that is closest to the level at which 35% of patients experience a DLT over the treatment period of seliciclib. The definition of a DLT and its target probability were agreed at a TRAFIC Investigators consensus meeting as comparable to the rate of sustained tolerated treatment on conventional synthetic DMARDs such as methotrexate [10].

Phase I will include a maximum of 21 patients. Up to seven cohorts of three participants each will be treated. A cohort size of three was chosen as it is logistical to manage across multi-centres with six planned suspension periods after each cohort to assess DLT. A cohort size of one would imply that the trial has to be suspended twenty times to assess DLT, which would make the trial unfeasibly long. Cohorts of three are a good compromise for the trial duration and allowing the model to update frequently with the accruing data. The number of doses was informed by the oncology experience which encompasses five dose levels of IMP: participants will receive either 200 mg, 400 mg, 600 mg, 800 mg or 1000 mg seliciclib daily for 4 consecutive days (one cycle) every week for 4 weeks. The prior estimate of MTD is 600 mg (dose level 3) of seliciclib (Table 1) but, to exercise caution, 400 mg seliciclib (dose level 2) is the starting dose. The design allows for de-escalation to a lower dose of 200 mg (dose level 1). The starting dose, a prior estimate of the MTD, dose range and schedule are based on healthy control data and oncology experience [7]. A sample size of 21 patients, from 7 cohorts, is sufficient to identify the expected dose (prior to the trial) from potential dose escalations from the starting dose, two de-escalation doses to the estimated dose, plus one additional confirmatory cohort at the final selected dose, resulting in 7 cohorts of 3 patients per cohort. The performance of the design was assessed via simulation.

**Table 1:** Prior probability that each Dose Level is the MTD for differing *σ*_*β*_, δ=0.06

Dose level	Prior SD of slope parameter β***,*** σ_**β**_		
	0.1	σ_β_^***LI***^ ***= 0.265***	√*1.34 =1.16*
**1 (200 mg)**	0.01	**0.20**	0.42
**2 (400 mg)**	0.22	**0.19**	0.05
**3 (600 mg)**	0.54	**0.22**	0.05
**4 (800 mg)**	0.22	**0.19**	0.05
**5 (1000 mg)**	0.01	**0.20**	0.42

Note: a large σ_β_ does not necessarily correspond to an uninformative prior for MTD level; √*1.34* is often chosen as the default value for CRM models. The least informative value of *σ*_*β*_ is one where there are almost equal prior probabilities (chance) that each dose level is the MTD across the 5 dose levels

The recommended dose (the dose with estimated DLT probability closest to the target of 35%) for each of the subsequent cohorts is determined using the CRM incorporating all of the accumulated DLT outcomes but for added safety, the design includes a restriction to prevent skipping of untested doses when escalating. Recruitment continues until either the maximum sample size is reached, the trial is stopped early due to unacceptable levels of DLT at the lowest dose or when there are four consecutive cohorts allocated to the recommended MTD (providing sufficient evidence that the MTD is reached). The two early stopping rules allow for early termination:

1. If there is a high probability (> 0.7) that the posterior probability of DLT at the lowest dose is greater than the target DLT rate of 35%, indicating that the lowest dose is too toxic.
2. If four consecutive cohorts (three patients in each cohort) have already been allocated at the current MTD, which would also be the recommended dose level for the next cohort if the trial continued.

**Fig. 1:**
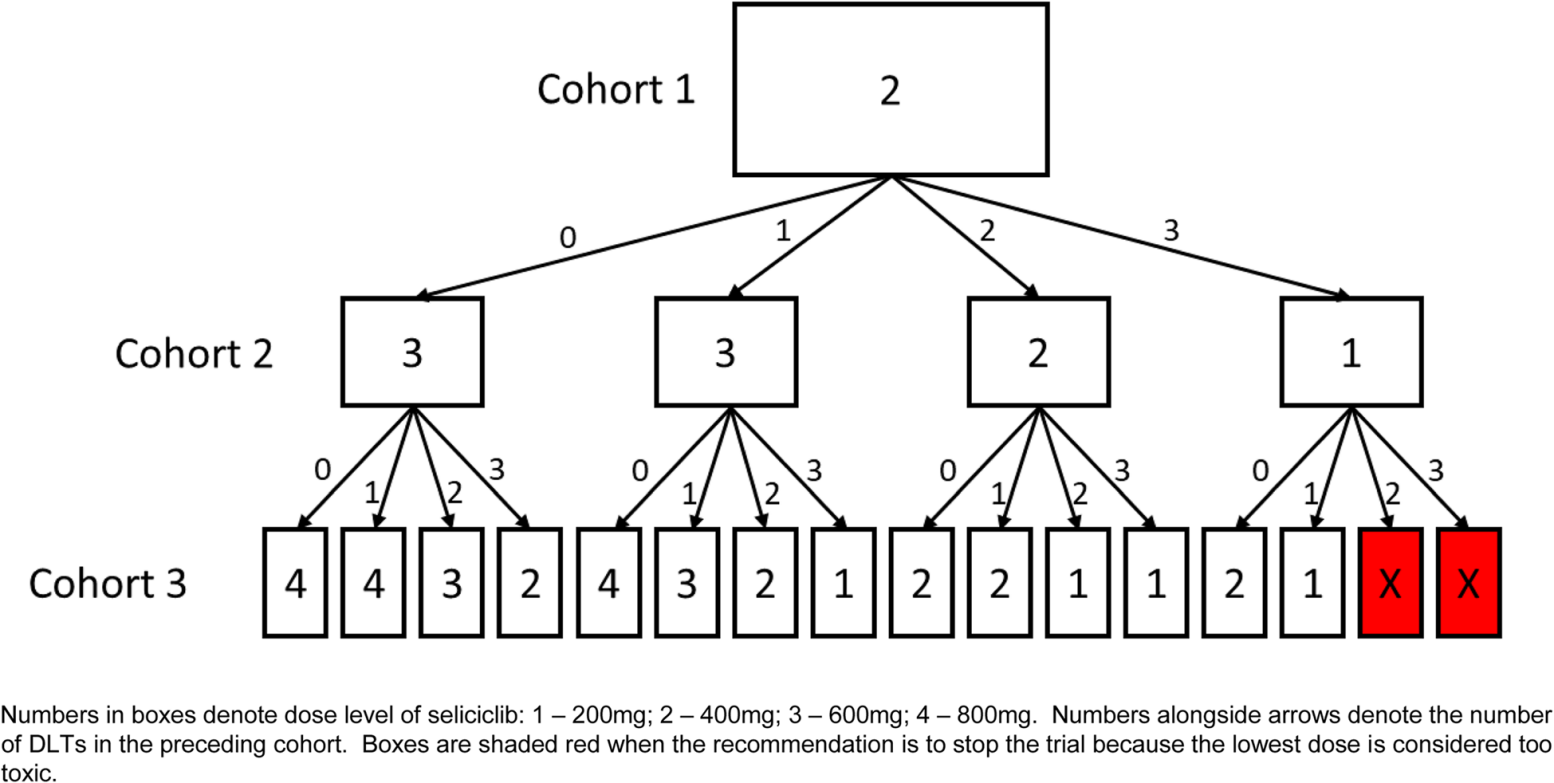
Dose transition pathways flow diagram for first three cohorts

The value of 0.7 was selected so that the design will recommend stopping early for excessive toxicity if we observe 2 or 3 DLTs out of the first 3 patients at the lowest dose level. This is exemplified in Fig. 1 which shows the dose transition pathways (DTP) for the first three cohorts. DTP are utilised (i) as a calibration tool to help evaluate potential designs and (ii) to visualise projected dose recommendations for clinical acceptance [11, 12].

In addition, DTP project possible pathways for all subsequent cohorts, allowing recruitment to proceed if the outcome of the final patient within the current cohort does not alter the dose recommendation for the next cohort. For TRAFIC, however, to avoid potential bias at trial conduct, only the next cohort is displayed. If implemented in full, this strategy can reduce the operational demands on the trial management group, including the trial statistician. Decisions to progress to the next cohort are considered by the TRAFIC Data Monitoring Committee (DMC) who make recommendations regarding the continuation of the trial.

At trial completion, the number of patients experiencing DLT at each dose level, together with the proportion of patients with DLT at that dose level, will be reported. The Bayesian posterior probability of DLT at each dose level (with 90% probability interval) will be reported graphically and in tabular form. The MTD will be reported as well as the posterior probability that the DLT rate at dose level 1 (200 mg) is greater than the target level of 35%. Secondary outcome measures, including pharmacodynamic (PD) biomarkers and pharmacokinetic (PK) parameters, will be presented descriptively and graphically.

### Continual reassessment method model details

The one-stage, one parameter Bayesian logistic dose-toxicity model used in TRAFIC is given by:

$$ \upvartheta \left(x,\upbeta \right)=\frac{\exp \left(3+{\mathrm{e}}^{\upbeta}x\right)}{1+\exp \left(3+{\mathrm{e}}^{\upbeta}x\right)}\kern2em \mathrm{for}-\infty <x<\infty, $$

where x is the scaled dose and ϑ(x, β) is the probability of a DLT at dose x [13]. The model parameter β is assumed to be random with prior distribution$\mathrm{N}\left(0,{\upsigma}_{\upbeta}^2\right)$. The Bayesian CRM model is completed by a specification of the prior probability of a DLT associated with the five test doses (the skeleton) and ${\upsigma}_{\upbeta}^2,$the prior variance of β. Early termination is allowed if there is a high probability that the posterior probability of DLT at the lowest dose is greater than the target DLT rate. Determining the posterior distribution of the DLT occurrence probability at the lowest dose requires the posterior distribution for β to be evaluated. After the first *n* patients, this is given by:

$$ \mathrm{p}\left(\upbeta\ |{\mathrm{X}}_{\mathrm{n}},{\mathrm{Y}}_{\mathrm{n}}\right)\propto \exp \left(\frac{-{\upbeta}^2}{2{\upsigma}_{\upbeta}^2}\right){\prod}_{\mathrm{i}=1}^{\mathrm{n}}\upvartheta {\left({\mathrm{x}}_{\mathrm{i}},\upbeta \right)}^{{\mathrm{y}}_{\mathrm{i}}}{\left(1-\upvartheta \left({\mathrm{x}}_{\mathrm{i}},\upbeta \right)\right)}^{1-{\mathrm{y}}_{\mathrm{i}}}, $$

where X_n_ = {x_1_, x_2_, …, x_n_} are the scaled doses and Y_n_ = {y_1_, y_2_, …, y_n_} are the DLT outcomes (0 or 1).

The posterior probability that the DLT occurrence probability at dose level 1 is greater than some rate ϑ_L_ is given by ${\int}_{-\infty}^{\upbeta_{\mathrm{L}}}\mathrm{p}\left(\upbeta\ |{\mathrm{X}}_{\mathrm{n}},{\mathrm{Y}}_{\mathrm{n}}\right)\mathrm{d}\upbeta$ where ${\upbeta}_{\mathrm{L}}=\log \left\{\frac{\log \left(\frac{\upvartheta_{\mathrm{L}}}{1-{\upvartheta}_{\mathrm{L}}}\right)-3}{{\mathrm{x}}_{\mathrm{D}1}}\right\}$ and x_D1_ is the scaled dose for dose level 1. The integral ${\int}_{-\infty}^{\upbeta_{\mathrm{L}}}\mathrm{p}\left(\upbeta\ |{\mathrm{X}}_{\mathrm{n}},{\mathrm{Y}}_{\mathrm{n}}\right)\mathrm{d}\upbeta$ is analytically intractable and so is obtained by numerical integration [14].

### Calibration of the continual reassessment method design

The Bayesian CRM model is calibrated (specification of the skeleton and prior variance of *β*) using the following algorithm of Cheung [13] which was implemented using a modified version of the R function *mtrials* part of the *dfcrm* R library [15]:

- For a given indifference interval half-width δ, the associated skeleton is obtained using the *dfcrm* function *getprior*. The indifference interval is an interval into which the DLT probability of the selected dose will eventually fall given a sufficiently large sample size.
- For each δ, the least informative prior standard deviation for the slope parameter β, ${\upsigma}_{\upbeta}^{\mathrm{LI}}\left(\delta \right)$, is determined. *σ*_*β*_ is defined to be least informative if the prior probability that a dose level is the MTD is approximately equal for all dose levels, that is, the distribution of the MTD (which depends on *σ*_*β*_) corresponds to the uniform distribution. Thus, ${\upsigma}_{\upbeta}^{\mathrm{LI}}$ is defined to be *σ*_*β*_ such that the SD of the MTD equals $\sqrt{\left({K}^2-1\right)/12}$, where K is the number of dose levels. Table 1 shows the effect of varying *σ*_*β*_ for TRAFIC when δ=0.06; a large σ_β_ does not necessarily correspond to an uninformative prior for the MTD dose level whereas ${\upsigma}_{\upbeta}^{\mathrm{LI}}$ does ensure almost equal prior probability that each of the five dose levels is the MTD.
- The pair {δ, σ^LI^_β_ (δ)} was chosen based upon the evaluation of the following performance measures: (i) the ability to correctly select the true MTD—the risk-adjusted average accuracy, A_N_ (weighting the probability of selecting a dose level by the absolute discrepancy between the true probability of toxicity at that dose level and the target probability of toxicity), and the unadjusted probability of correctly selecting the true MTD; (ii) optimal allocation defined as the mean proportion of patients treated within one dose level of the true MTD; (iii) the mean proportion of patients treated at an overdose (a dose above the true MTD); and (iv) the mean number of patients treated [13].

### Operating characteristics of the continual reassessment method design

The performance measures in (c) above were estimated by Monte Carlo simulation: 20,000 trials were simulated for different values of δ (0.02 to 0.20 in steps of 0.01). Trials were simulated assuming the underlying probabilities of DLT (Table 2). These are the appropriate plateau calibration configurations with five test dose levels when the target DLT rate is 35% [13]. The performance of the design also was assessed via simulation under several clinically relevant scenarios where, firstly, the true probability of DLT follows the skeleton chosen for TRAFIC and, secondly, the MTD is assumed to occur at each of the remaining four dose levels.

**Table 2:** Probability of DLT at each of five dose levels for five different dose-toxicity curves. Plateau calibration configuration curves for target DLT probability θ=0.35 and five test dose levels

Curve	Dose level				
	1	2	3	4	5
1	**0.35**	0.52	0.52	0.52	0.52
2	0.21	**0.35**	0.52	0.52	0.52
3	0.21	0.21	**0.35**	0.52	0.52
4	0.21	0.21	0.21	**0.35**	0.52
5	0.21	0.21	0.21	0.21	**0.35**

**Fig. 2:**
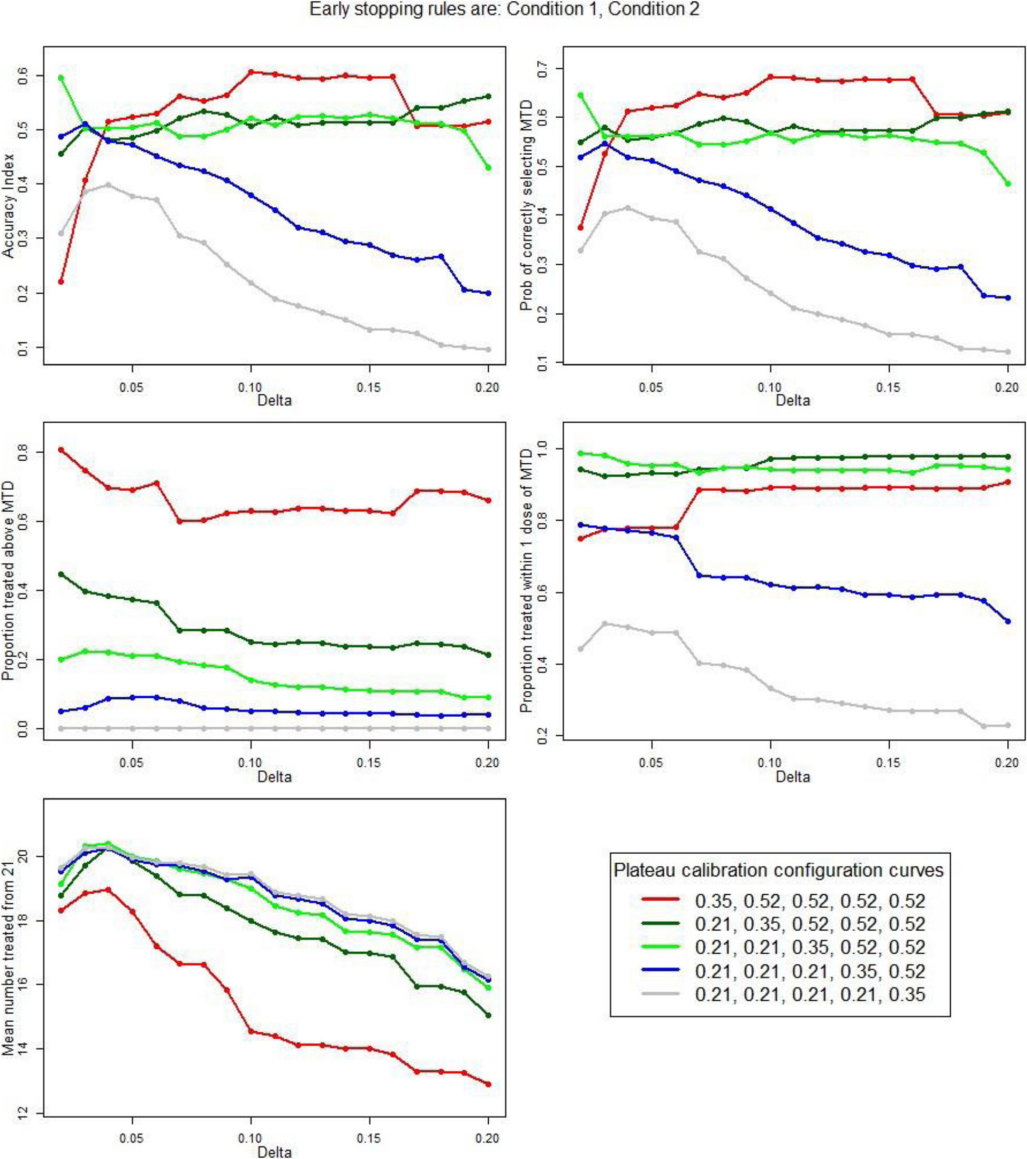
Performance measures against the indifference interval half-width, δ, for each of the plateau calibration curves. Early stopping rules are condition 1 and 2

Dose level 1 (200 mg) was thought unlikely to be efficacious; hence, calibration of the CRM focused on achieving (1) good accuracy, optimal allocation and minimising the proportion of patients treated at an overdose when the true simulated MTD was at dose levels 2 to 5 and (2) minimising the number of patients treated when the true MTD was at dose level 1. Performance measures implementing the early stopping rules 1 and 2 are plotted against δ (Fig. 2) for each of the calibration curves (Table 2).

**Fig. 3:**
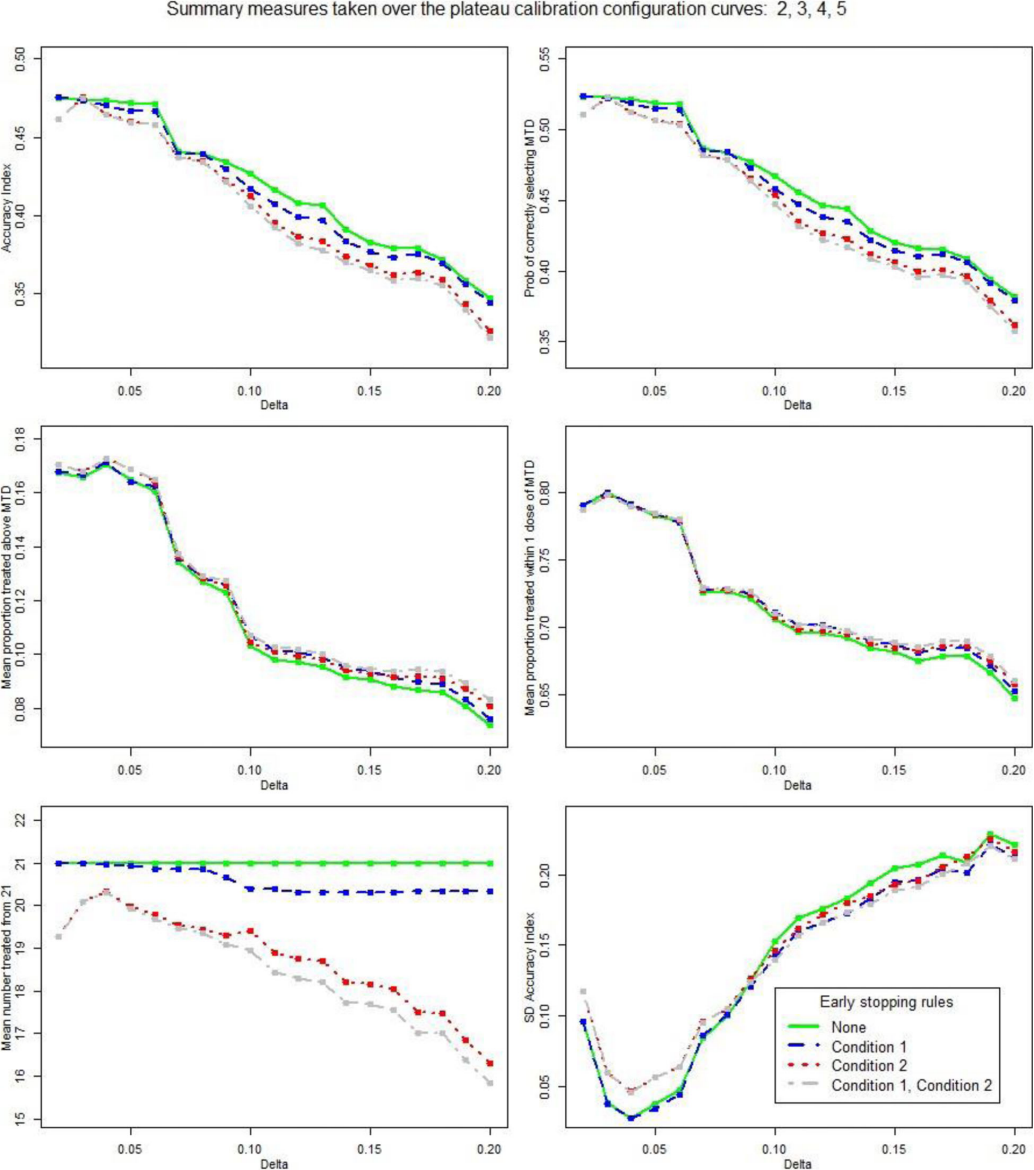
Summary statistics for the performance measures taken over the plateau calibration curves 2, 3, 4 and 5. Performance measures are plotted against the indifference interval half-width, δ, and shown according to various stopping criteria

The mean of the performance measures taken over the calibration curves 2, 3, 4 and 5, i.e. when the true simulated MTD is at dose levels 2, 3, 4 and 5 are given in Table 3 and Fig. 3. The performance measures (1) accuracy index A_N_ [13], (2) the unadjusted probability of selecting the true MTD, (3) the mean proportion of patients treated at an overdose and (4) the mean proportion of patients treated within 1 dose of the true MTD, all show a similar relationship with δ. As δ increases, the measures remain fairly constant until δ=0.06 when they drop significantly. Except at very low levels of δ, the mean number of patients treated decreases as δ increases, particularly so when the simulated true MTD is dose level 1. The variation in A_N_ over the calibration curves 2, 3, 4 and 5, SD (A_N_), is smallest when δ=0.04 and remains low until δ exceeds 0.06 when it increases rapidly. This variation should be low to ensure reasonable performance across dose levels 2 to 5, as opposed to a good performance at some doses and poor performance at others. Given the objective of achieving a balance between good performance when the true MTD is at dose levels 2 to 5 and minimising the number of patients treated when the true MTD is at dose level 1, δ=0.06 was selected.

**Table 3:** Summary statistics for the performance measures over the plateau calibration curves 2, 3, 4 and 5 for increasing levels of the indifference interval half-width δ. δ=0.06 is in bold; this is the level chosen for TRAFIC

δ	Accuracy index A_N_	Probability of correctly selecting MTD	Mean proportion above MTD	Mean proportion within 1 dose of MTD	Mean number treated	SD A_N_
**0.02**	0.46	0.51	0.17	0.79	19.3	0.12
**0.03**	0.48	0.52	0.17	0.80	20.1	0.06
**0.04**	0.46	0.51	0.17	0.79	20.3	0.05
**0.05**	0.46	0.51	0.17	0.78	19.9	0.06
**0.06**	**0.46**	**0.50**	**0.16**	**0.78**	**19.7**	**0.06**
**0.07**	0.44	0.48	0.14	0.73	19.5	0.09
**0.08**	0.43	0.48	0.13	0.73	19.4	0.11
**0.09**	0.42	0.46	0.13	0.73	19.1	0.12
**0.10**	0.41	0.45	0.11	0.71	18.9	0.14
**0.11**	0.39	0.43	0.10	0.70	18.4	0.16
**0.12**	0.38	0.42	0.10	0.70	18.3	0.17
**0.13**	0.38	0.42	0.10	0.70	18.2	0.17
**0.14**	0.37	0.41	0.10	0.69	17.7	0.18
**0.15**	0.36	0.40	0.09	0.69	17.7	0.19
**0.16**	0.36	0.40	0.09	0.69	17.6	0.19
**0.17**	0.36	0.40	0.09	0.69	17.0	0.20
**0.18**	0.36	0.39	0.09	0.69	17.0	0.21
**0.19**	0.34	0.38	0.09	0.68	16.4	0.22
**0.20**	0.32	0.36	0.08	0.66	15.8	0.21

Simulations to assess the chosen level of δ for various dose-toxicity relationships (Table 4) assume both the true probability of DLT follows the skeleton chosen for TRAFIC (simulation 1) and where the assumed true MTD across the five dose levels is varied (simulations 2–6). The performance of the final proposed CRM design is promising as indicated by the accuracy index A_N_ which is above 0.90 for each simulation scenario. The final design parameters are summarised in Table 5.

**Table 4:** Performance measures for simulated trials

Simulation scenario	Assumed probability of DLT					Accuracy index A_N_	Probability of correctly selecting MTD	Mean proportion above MTD	Mean proportion within one dose of MTD	Mean number treated
	Dose level									
	1	2	3	4	5					
1	0.14	0.23	0.35	0.47	0.57	0.91	0.50	0.22	0.95	19.7
2	0.35	0.40	0.50	0.60	0.70	0.93	0.36	0.83	0.67	18.4
3	0.15	0.35	0.40	0.50	0.60	0.92	0.45	0.46	0.88	19.3
4	0.05	0.15	0.35	0.50	0.60	0.93	0.62	0.25	0.97	20.1
5	0.05	0.15	0.25	0.35	0.60	0.92	0.50	0.08	0.78	20.0
6	0.05	0.10	0.20	0.30	0.35	0.93	0.34	0.00	0.53	20.2

**Table 5:** Final trial design parameters

**Clinical parameters**	**DLT definition**: cessation of IMP due to AE/AR **Dose set**: 200, 400, 600, 800, 1000 mg daily **Starting dose**: 400 mg **Fixed or variable sample size**: fixed, n=21 **Target level of DLT**: 35% **Cohort size**: 3
**Model specification parameters**	**Model for dose-toxicity curve**: Bayesian logistic regression **Initial MTD prior**: 600 mg **One-stage or two-stage design**: One **Estimation approach**: Bayesian **Skeleton**: (0.14, 0.23, 0.35, 0.47, 0.57) **Model**: one parameter Bayesian model with prior distribution for slope N (μ = 0, **σ** = 0.265)
**Practical considerations**	**Skipping doses**: No **Early stopping rules**: Yes i) High probability (> 0.7) that posterior probability of DLT at lowest dose is greater than target DLT of 35%, indicating that the lowest dose is too toxic ii) 4 consecutive cohorts allocated MTD, providing sufficient evidence MTD is reached

### Phase II Fleming A’Hern design

Phase II is a single-arm, single-stage early phase trial based on a Fleming-A’Hern design [16] recruiting a total of 18 patients at the recommended phase II dose who provided written informed consent, with baseline and 12-week outcome data. Efficacy is assessed via clinical assessments, analysis of synovial biopsies and contrast-enhanced MRI of an affected hand and wrist. The primary outcome measure is a composite response rate at 12 weeks defined as achieving two of the following three criteria: (i) EULAR moderate response or ACR20 response, (ii) histological reduction in macrophage number in the sub lining layer of the synovium ≥ 20% and (iii) reduction of Rheumatoid Arthritis MRI Scoring System score, on MRI, of ≥ 0.5 units or osteitis score of ≥ 0.2 units. Secondary outcome measures are as for phase I and, additionally, changes in PD biomarkers in synovial tissue and response rate after 1, 2, 3, 6 and 9 weeks of therapy.

Response rate will be calculated as the total number of patients responding as a proportion of all patients who start treatment. Any patients who are not assessable at 12 weeks will be classed as a non-responder. Individual components of the composite response outcome will be reported descriptively. Adverse events will be reported as the number of patients experiencing an event as a proportion of the total number of patients starting treatment, reported descriptively. PK parameters and PD biomarkers will be presented graphically.

The Fleming A’Hern design assumes a composite response rate to reject seliciclib (p0) < 25% and a response rate to investigate seliciclib further (p1) > 50%. The justification to investigate seliciclib further is based on observing a critical minimum number of responses, as specified in the statistical analysis plan. As an early phase trial, the error levels are inflated but restricted to an acceptable level of < 15% α (type 1) and < 20% β (type 2). With these stated parameters, the target recruitment for phase II is calculated as 18 patients.

At the conclusion of phase II, the Fleming A’Hern design would indicate no further investigation is warranted if the observed number of clinical responses is less than the critical number, retained in the statistical trial master file. As this is the first trial investigating seliciclib in this indication (as a repurposed drug with a novel mechanism of action), the decision to collate further phase 2 evidence will also be based on clinically relevant PD biomarkers and PK parameters, since the TRAFIC trial management group would not want to reject a potentially active drug which has not achieved pragmatic clinical measures of efficacy. Specifically, in terms of synovial PD biomarkers: (i) TaqMan low-density transcriptional arrays incorporating genes of interest relevant to fibroblast biology, inflammation, cell cycle and apoptosis applied to mRNA extracted from whole synovial tissue; (ii) PD effects of seliciclib on the synovial fibroblast; and (iii) markers of cell proliferation, such as Ki-67.

## Discussion

The TRAFIC trial is the first reported dose-finding trial using a model-based design in inflammatory disease. It is a non-commercial, multi-centre, phase I/II early phase Bayesian adaptive model-based dose-finding trial designed to determine the safety, tolerability and efficacy of seliciclib as an addition to existing therapy in patients with RA. There remains a clinical need for novel therapeutics or drug repurposing in rheumatoid arthritis given sub-optimal responses to all available therapies in a significant proportion of patients. Moreover, the field needs to be able to make rapid decisions with new modes of action as early as possible to optimise trial recruitment. Efficient designs are required to minimise patient exposure pending estimation of safety. TRAFIC is a rolling phase I/II trial bringing efficiencies in site activity, patient numbers, external monitoring and costs, minimising some of the challenges when conducting early phase trials.

There are demonstrated advantages of model-based designs for determining maximum tolerated dose in dose-finding studies, but uptake has been slow in disciplines outside of cancer trials. The statistical calibration and implementation of the TRAFIC trial demonstrate that it is possible to design, fund and implement a model-based continual reassessment method phase I trial in a non-cancer population within research funding and regulatory constraints.

The design allows learning of information from cancer patients to inform the TRAFIC trial design parameters, specifically starting dose, prior estimate of the maximum tolerated dose, dose range and schedule. The Bayesian continual reassessment method allows all available accumulated data to be utilised to inform decision-making regarding dose escalation, de-escalation or retention through progressive cohorts. The Bayesian continual reassessment method also allows prior data to be utilised in the prior distribution, although TRAFIC conservatively opted for a least informative skeleton. Simulation allows the operating characteristics of the proposed adaptive design to be assessed and the approach in TRAFIC incorporates two stopping rules. At the design stage, the use of the dose transition pathways (DTP) encourages closer engagement with the clinical investigators, trials management team and statisticians and improves understanding of how such a model may work in practice. Such discussions are particularly helpful to gather and incorporate vital clinical opinions in the development and calibration of the model. Both simulations and DTP play an instrumental role in the successful implementation of this method, which is considerably more complex than a simple rule-based design such as the 3 + 3. The phase II design of TRAFIC demonstrates that even small, single-arm trials can have an underlying statistical basis for decision-making and interpretation, often discounted.

Operationally, identified barriers including longer set-up time for statistical simulation and trial management implementation are beginning to be addressed through NIHR and UKCRC training for both clinical and statistical teams alike [17]. The TRAFIC trial design was supported through NIHR UKCRC CTU infrastructure funding prior to protocol development being funded by MRC. There still remains a need for unified support from regulators and journal editors to promote more accurate dosing. Other operational considerations include managing recruitment to small, fixed cohort sizes across multiple sites, potential to skip doses and stop early and implementing efficient Data Monitoring Committee decision-making to allow smooth movement between cohorts. Implementation of a dose transition pathway allows a transparent, graphical interpretation of the trial design to facilitate upfront discussion and decision-making with clinical teams. It can enable an efficient look ahead strategy although any associate bias in doing so must be considered. The dose transition pathway can provide understanding and guidance to the Data Monitoring Committee and is recommended to facilitate upfront discussion and consensus on trial conduct.

The rolling design recruiting patients seamlessly from phase I to phase II allows site activity and momentum to be retained and also brings trial conduct efficiencies including internal site monitoring and external data monitoring activities, with obvious cost savings. The rolling design allows continuity for clinical, trial and monitoring teams maintaining relationships and consistently high quality across trial management activities across phases. A rolling design has the potential advantage to allow patients in the final cohort in phase 1 to be retained as the first patients recruited to phase 2, dependent upon the dose in the final cohort. Altogether, this approach to the trial design is attractive to clinical, trial and site staff and efficient in-patient recruitment when conducting challenging early phase trials. The TRAFIC trial demonstrates that small phase I/II trials can have an underlying statistical basis for decision-making and interpretation. TRAFIC will follow the CONSORT reporting recommendations to describe a transparent statistical design reflecting relevant clinical opinion and decision-making.

The re-purposing of drugs across diseases requires re-dosing and safety assessment and as the potential for repurposing increases, so does the potential to conduct trials with model-based designs to more accurately estimate optimal dosing. The TRAFIC trial is the first reported dose-finding trial of re-purposed treatment using a model-based design in inflammatory disease, conducted within the academic environment within research funding and regulatory constraints.

## Trial status

Protocol v11.00 21-March-2019. Open to recruitment: March-2015, recruitment completion expected June 2021.

## Data Availability

The final trial dataset will be held by the Newcastle University. Data sharing requests may be submitted following the publication of results.
